# CircUBE2Q2 promotes differentiation of cattle muscle stem cells and is a potential regulatory molecule of skeletal muscle development

**DOI:** 10.1186/s12864-022-08518-4

**Published:** 2022-04-06

**Authors:** Rui-Men Zhang, Yu Pan, Chao-Xia Zou, Qiang An, Juan-Ru Cheng, Peng-Ju Li, Zi-Hua Zheng, Yan Pan, Wan-You Feng, Su-Fang Yang, De-Shun Shi, Ying-Ming Wei, Yan-Fei Deng

**Affiliations:** 1 State Key Laboratory for Conservation and Utilization of Subtropical Agro-bioresources, Animal Reproduction Institute, Guangxi University, Nanning, 530004 Guangxi China; 2 Guangxi Agricultural Vocational University, Nanning, 530007 Guangxi China; 3 International Zhuang Medical Hospital Affiliated to Guangxi University Chinese Medicine, Nanning, 530000 Guangxi China

**Keywords:** Cattle, Muscle stem cells, circRNAs, Proliferation, Differentiation

## Abstract

**Background:**

The growth and development of muscle stem cells (MuSCs) are significant events known to affect muscle plasticity, disease, meat production, and meat quality, which involves the types and functions of mRNA and non-coding RNA. Here, MuSCs were cultured from Guangxi fetal cattle. RNA sequencing was used to analyze the RNA expression of mRNA and non-coding RNAs during the cell proliferation and differentiation phases.

**Results:**

Two thousand one hundred forty-eight mRNAs and 888 non-coding RNAs were differentially expressed between cell proliferation and differentiation phases, including 113 miRNAs, 662 lncRNAs, and 113 circRNAs. RT-qPCR verified the differential expression levels of mRNAs and non-coding RNAs, and the differentially expressed circUBE2Q2 was subsequently characterized. Expression profile analysis revealed that circUBE2Q2 was abundant in muscle tissues and intramuscular fat. The expression of cricUBE2Q2 was also significantly upregulated during MuSCs myogenic differentiation and SVFs adipogenic differentiation and decreased with age in cattle muscle tissue. Finally, the molecular mechanism of circUBE2Q2 regulating MuSCs function that affects skeletal muscle development was investigated. The results showed that circUBE2Q2 could serve as a sponge for miR-133a, significantly promoting differentiation and apoptosis of cultured MuSCs, and inhibiting proliferation of MuSCs.

**Conclusions:**

CircUBE2Q2 is associated with muscle growth and development and induces MuSCs myogenic differentiation through sponging miR-133a. This study will provide new clues for the mechanisms by which mRNAs and non-coding RNAs regulate skeletal muscle growth and development, affecting muscle quality and diseases.

**Supplementary Information:**

The online version contains supplementary material available at 10.1186/s12864-022-08518-4.

## Introduction

The global consumption of beef is increasing on a yearly basis, and it is an indispensable food in the lives of people in modern society [1]. This is reflected in that the beef cattle industry is becoming more and more critical to modern agriculture [2, 3]. Muscle diseases are one of the severe diseases plaguing humankind [4]. Hence, finding the mechanism of controlling muscle mass is necessary to treat muscle injury and atrophy and study muscle growth regulation.

Muscle tissue plays a key role in regulating the body’s metabolism and homeostasis, accounting for about 40–60% of the weight of adult animals [5], which not only determines the levels of meat production performance but also has an essential impact on meat quality [6]. Failure to develop skeletal muscles before birth can lead to embryonic lethality. Failure to repair or maintain skeletal muscle regeneration after birth can reduce the quality of life or even death. In humans with muscular dystrophy, cachexia and sarcopenia are examples of harmful types of muscular disorders [7]. In ruminant skeletal muscle tissues, there is a group of myoblasts-muscle stem cells [8, 9], which is the primary source of skeletal muscle formation and regeneration. Muscle-derived stem cells have the potential capability of differentiation and proliferation and are also a cell model for research into muscle development. Under certain conditions, as an essential type of myoblasts, muscle stem cells (MuSCs) can be activated, which subsequently proliferate and differentiate into skeletal muscle or other types of cells, such as fat-like cells, osteoblasts, and nerve cells.

The growth and development of animal skeletal muscles involve several complex molecular processes. Many functional genes participate in their regulation, and these actions and the signaling pathways constitute a distinct regulatory network [10]. For example, myogenic regulatory factors (MRFs) [11], myocyte enhancer factor 2 (*MEF2)*, [12], *PAX3* / *PAX7* [13], and myostatin (*MSTN*) [14] are considered as core genes involved in skeletal muscle growth and development.

Non-coding RNAs such as microRNAs (miRNAs), long non-coding RNAs (lncRNAs), and circular RNAs (circRNAs) have been discovered in mammalian genome transcription, and these RNA molecules play key roles in different biological processes and diseases. Compared to miRNA, research into lncRNA and circRNA is still in their infancy [15]. So far, the roles of lncRNAs and circRNAs molecules in beef cattle skeletal muscle development in agricultural animals have not been explored fully.

Muscle development-related miRNAs have been extensively studied and characterized [16]. Muscle-specific miR-1, miR-206, and miR-133 are necessary for muscle development and function. Overexpression of miR-1 or miR-206 could accelerate primary muscle differentiation, and overexpression of miR-133 promoted myoblast proliferation but inhibited the differentiation process [17]. LncRNAs were initially considered to be transcriptional noise. Nevertheless, LncRNA-linc-MD1 was identified as a role in myogenesis, and lncRNAs were involved with muscle formation. In particular, lncRNA H19 [18], lnc-AK143003 [18, 19], and lnc-133b [20] had specific roles in this process. A large number of critical regulatory factors contributed to the role of lncRNAs in the muscle regulatory network [5].

To date, the identification of covalently closed circRNAs has reshaped the perspectives of the linear RNA world. CircRNA is produced by reverse splicing a pre-mRNA of a gene exon in eukaryotic cells. It has a closed-loop structure, making it less susceptible to degradation by exonuclease RNase R, thus making it more stable than other types of RNA. Studies have found that there are four main functions of circRNAs. First, circRNAs combine with proteins to form RNA-protein complexes that act on target genes. For example, circ-FOXO3 can form a circ-FOXO3-CDK2-P21 ternary complex with cyclin-dependent kinase2 (CDK2) and P21 to inhibit the cell cycle [21].

Furthermore, circRNAs can be used as miRNA sponges to compete with mRNA in the cytoplasm for binding to miRNA, thereby regulating gene expression [22]. CiRS-7 (miR-7 circular RNA sponge), circMYBPC1 was a typical representative of this type of circRNA [23]. Likewise, circRNAs can encode proteins, and circ-ZNF609 translation produces proteins involved in muscle growth and development [24]. Moreover, circRNAs can regulate gene transcription [25]. For instance, circFUT10 and some muscle-derived differentiation factors regulate the growth and development of skeletal muscle synergistically [17]. As their expression increased, MyoD, MyoG, and MyHC at the mRNA and protein levels also increased, promoting myotube formation. The role of these circRNAs is to add another level of control to the regulation of cellular biological processes within the RNA field [26].

In this study, we used RNA-seq technology to determine the RNA expression characteristics of the proliferation and differentiation phases of cattle MuSCs, especially concerning the regulatory function of circRNAs on muscle stem cell development. It is envisaged that these results will provide new clues for further elucidating the mechanisms whereby mRNAs and non-coding RNAs regulate muscle growth and development.

## Results

### Variations of phenotypic characteristics and markers during proliferation and differentiation of Guangxi cattle MuSCs

**Fig. 1:**
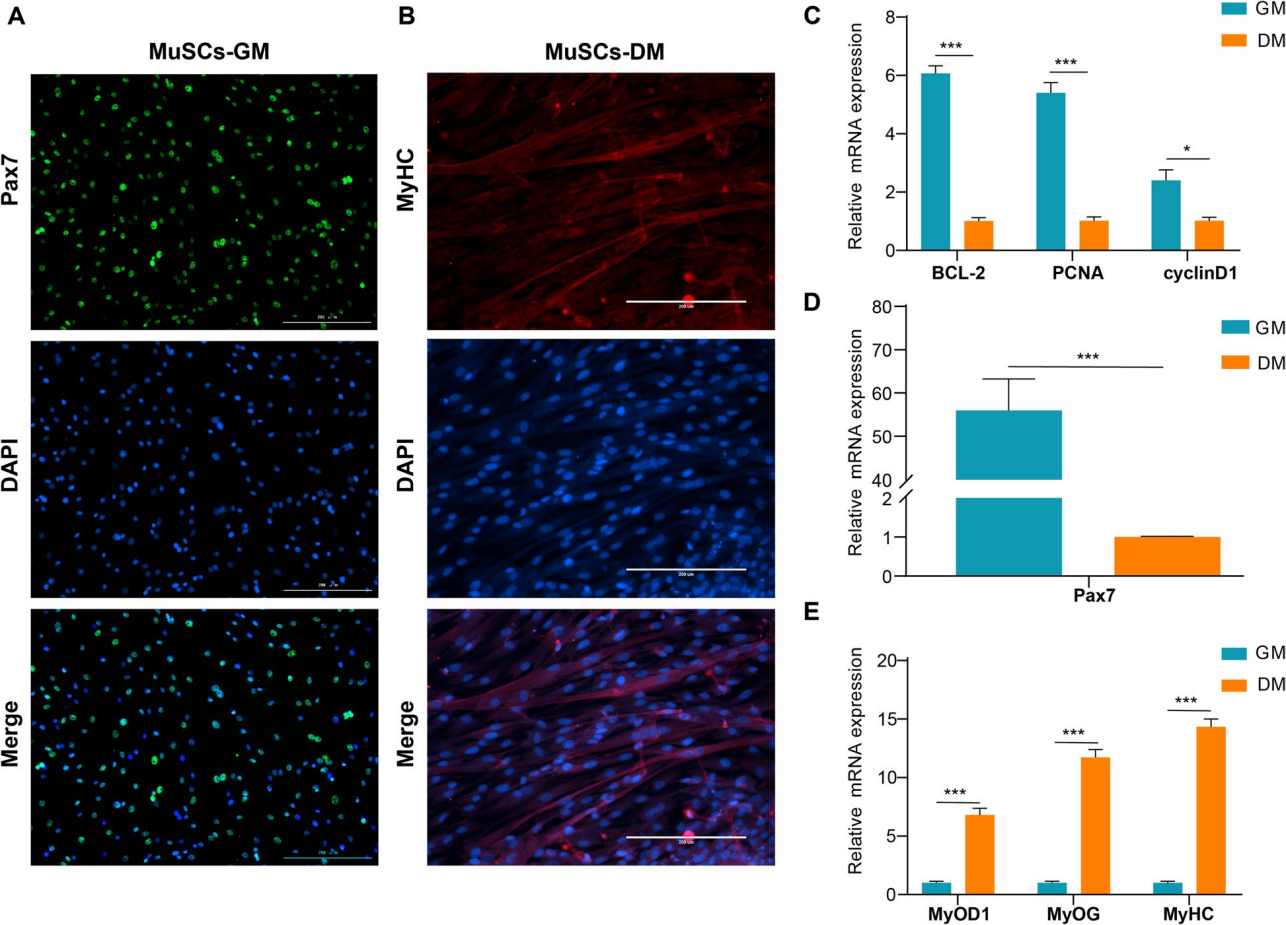
Analysis of the characteristics of Guangxi cattle MuSCs during proliferation and differentiation. **A** MuSCs proliferation [25] were detected by immunofluorescence of Pax7 and observed under a fluorescence microscope. **B** MuSCs differentiation (DM) was detected by immunofluorescence of MyOD1 and observed under a fluorescence microscope. **C**-**D** Measurement of BCL-2, PCNA, cyclin D1, and Pax7 mRNA expression levels by RT-qPCR. **E** Measurement of MyOD1, MyOG, and MyHC mRNA expression levels by RT-qPCR. Values are means ± SEM for three individual animals. ∗∗ *P* < 0.01. Scale bars = 200 μm

A combined digestion method consisting of type I collagenase and trypsin was used to obtain Guangxi cattle fetal-derived MuSCs (a beef cattle breed found in southern China). This cell type was similar to fibroblasts and was spindle-shaped in appearance. These cells had good proliferation capacity, which is subsequently referred to as the proliferation phase (GM samples) of MuSCs (Supplemental file 1, Fig. S1A-Fig. S1C). The marker of early proliferating myoblasts was paired homeobox transcription factor *Pax7,* and Pax7-positive cells up to 90% in MuSCs, suggesting these cells were characteristics of myogenic precursor cells (Fig. 1A). In addition, when the medium was replaced with DM, the number of myotubes increased on the fifth day. The myotube fusion became more obvious, referred to as the differentiation phase (DM samples) of MuSCs (Supplemental file 1, Fig. S1D). One of the muscle marker molecules, MyHC, was enriched in the DM of MuSCs (Fig. 1B). Subsequently, RT-qPCR showed that the expression of BCL-2, PCNA, CyclinD1, and Pax7 in the GM of MuSCs was significantly higher than that seen in the DM (Fig. 1C, D). On the other hand, the expression levels of MyoD1, MyoG, and MyHC increased significantly in DM of MuSCs (Fig. 1E).

### Analysis of RNA expression during proliferation and differentiation of MuSCs

**Fig. 2:**
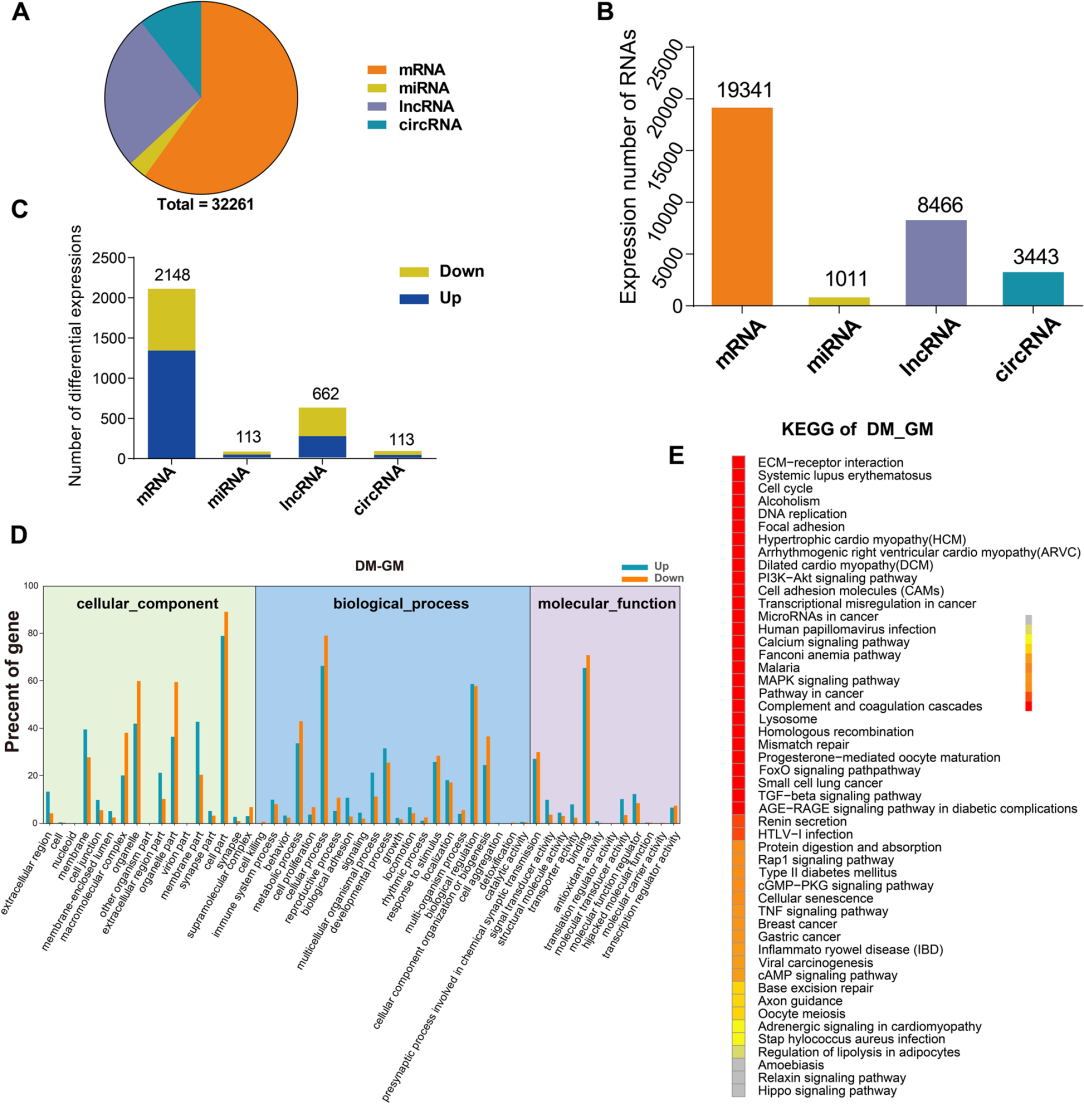
Analysis of the RNA expression profiles during proliferation and differentiation of cattle MuSCs. **A**, **B** Identification and quantitative distribution of mRNAs, miRNAs, lncRNAs, and circRNAs of MuSCs. **C** The statistics of differentially expressed mRNAs, miRNAs, lncRNAs, and circRNAs, including the upregulated and downregulated molecules (fold-change≥1.0, * *P* < 0.05). **D**, **E** The GO and top 50 KEGG terms of genes from differentially expressed mRNAs are shown

To identify MuSCs-related RNAs, we used GM (*n* = 3) and DM (n = 3) samples of MuSCs for RNA sequencing. Differential expression of mRNAs, miRNAs, lncRNAs, and circRNAs were then analyzed (Supplemental file 1, Fig. S2). Analysis of sequencing data revealed that 19,341 coding RNAs (mRNAs) and 12,920 non-coding RNAs were involved in the proliferation and differentiation of muscle stem cells, including 1011 miRNAs, 8466 lncRNAs, and 3443 circRNAs (Fig. 2A, B).

Heatmaps and volcano plots were also performed for mRNAs, lncRNAs, miRNAs, and circRNAs for MuSCs (fold-change ≥1.0, *P* < 0.05) (Supplemental file 1, Fig. S3). Two thousand one hundred forty-eight differentially encoded mRNAs and 888 non-coding RNAs, including 113 miRNAs, 662 lncRNAs, and 113 circRNAs (Fig. 2C). Compared with the GM samples of MuSCs, 1363 mRNAs (63.45%), 57 miRNAs (50.44%), 284 lncRNAs (42.90%) and 47 circRNAs (41.59%) were upregulated, while 785 mRNAs (36.55%), 56 miRNAs (49.56%), 378 lncRNAs (57.10%) and 66 circRNAs (58.41%) were downregulated in the DM samples of MuSCs (Supplemental file 2, Table S1). Further analysis of the circRNA sequencing data showed that the vast majority were exon-type circRNAs (2 to 6 exons), and some circRNAs were formed by one or more than 7 exons. The number of circRNAs produced by different chromosomes was also different. In this study, the numbers of circRNAs on chromosomes 6 and 22 were the largest, and the number on the X chromosomes was the smallest (Supplemental file 1, Fig. S4).

### Signal pathway enrichment and validation analysis of differentially expressed mRNAs during proliferation and differentiation of Guangxi cattle MuSCs

GO and KEGG enrichment analysis was performed on mRNAs involved in the regulation of differentiation, transformation, and maturation of MuSCs to predict the functions and mechanisms of miRNAs, lncRNAs, and circRNAs. The pathway analysis results of differentially expressed mRNAs showed that the GO was analyzed and annotated into differential genes of three main categories: biological processes, cellular components, and molecular functions (Fig. 2D). In addition, KEGG pathway enrichment analysis was employed to understand further the biological functions and molecular interactions of these differentially expressed mRNAs while assuming that the identified pathways may be involved in the development and growth of cattle skeletal muscle. We found 311 pathways to be enriched, and the top 50 most significant terms were uncovered, included biological processes such as ECM receptor interaction, cell cycle, DNA repair, and cell autophagy (Fig. 2E).

Based on the expression levels of differentially expressed genes, we screened a batch of highly expressed candidate research molecules such as *ZBTB16*, miR-133a, MSTRG.146272, and circLOM7 (bta-circ-0001851). Twenty four differentially expressed RNA molecules were randomly selected for RT-qPCR verification (Supplemental file 1, Fig. S9), including 7 mRNAs (*MYF6*, *IGFN1*, *MYF5*, *SGCA*, *MYEF2*, *KLF5,* and *COPB1*), 3 miRNAs (miR-206, miR-133a, and miR-199a-3p), 2 lncRNAs (MSTRG.146272 and MSTRG.19232) and 12 circRNAs (Table 1). After comparing the results with the RNA-seq data, we found that these genes had similar expression trends compared to RT-qPCR data. There were strong consistencies between the data obtained.

**Table 1:** Information regarding the circRNAs used in RT-qPCR in this study

CircRNA	Genomic position	Host gene	CircRNA ID
CircMAP4	NC_037349.1:51861172–51,868,891	MAP4	Bta_circ_0002890
CircCOPB1	NC_037342.1:38243200–38,247,001	COPB1	Bta_circ_0002163
CircRBM33	NC_037331.1:117404934–117,413,196	RBM33	Bta_circ_0000567
CircLMO7	NC_037339.1:50842015–50,873,294	LMO7	Bta_circ_0001851
CircKLF7	NC_037329.1:95427089–95,427,719	KLF7	Bta_circ_0000362
CircTAF4	NC_037340.1:55143763–55,148,383	TAF4	Bta_circ_0001969
CircUBE2Q2	NC_037348.1:31148563–31,167,136	UBE2Q2	Bta_circ_0002760
CircXDH	NC_037338.1:14186821–14,195,067	XDH	Bta_circ_0001640
CircVPS33A	NC_037344.1:53132830–53,144,656	VPS33A	Bta_circ_0002390
CircMYEF2	NC_037337.1:62229697–62,234,492	MYEF2	Bta_circ_0001547
CircRRAS2	NC_037342.1:38431604–38,432,981	RRAS2	Bta_circ_0002164
CircANLN	NC_037331.1:60800564–60,802,741	ANLN	Bta_circ_0000633

### Characteristics of CircUBE2Q2 in MuSCs and tissues

**Fig. 3:**
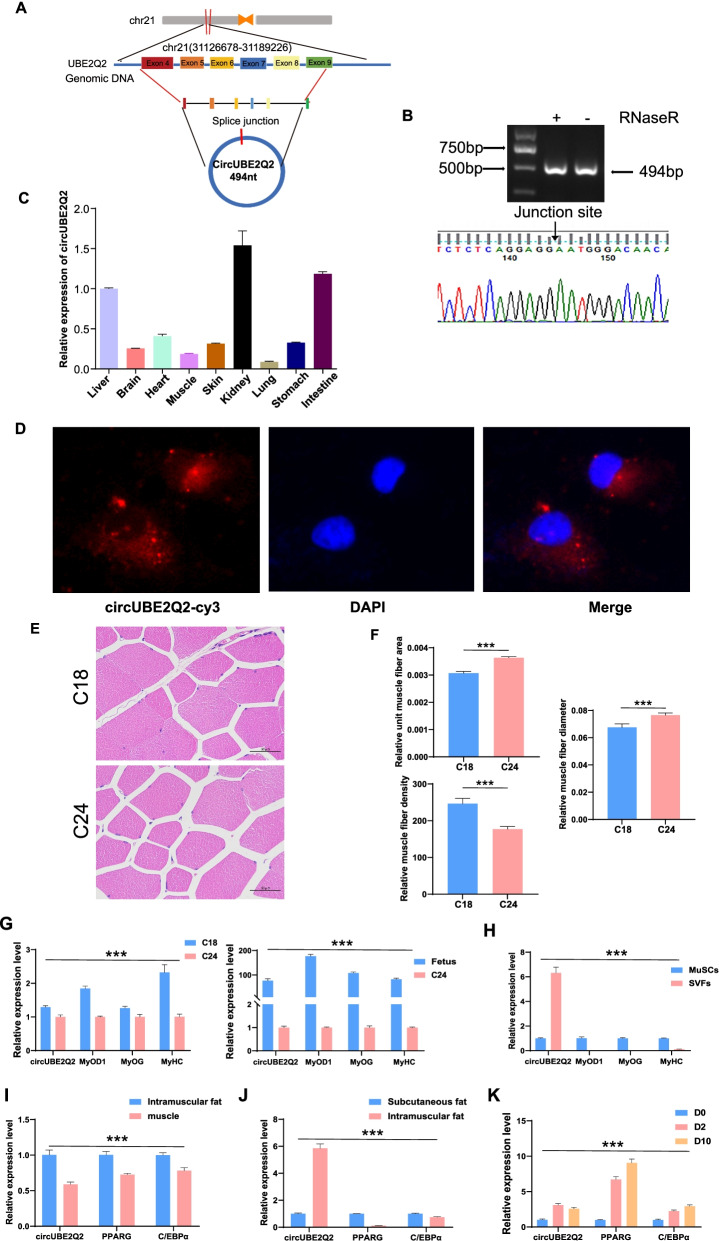
Expression characteristics of circUBE2Q2 in cells and tissues. **A** CircUBE2Q2 formed by the circularization of exons 4–9 of UBE2Q2 gene shearing, and the length of circUBE2Q2 was found to be 494 bp. The red arrow indicates the back splice site. **B** CircUBE2Q2 was examined by RT-qPCR after treatment with RNase R or mock in total RNAs samples derived from MuSCs. The circular junction of circUBE2Q2 was identified using divergent primers using Sanger sequencing. **C** The expression levels of circUBE2Q2 in different tissues of embryonic cattle. **D** The fluorescent in situ hybridization results showed that circUBE2Q2 was mainly located in the cytoplasm. Magnification 73x. **E** HE staining of muscle tissue of Guangxi cattle at 18 (C18) and 24 (C24) months of age. **F** The analysis of muscle fiber density, diameters, and area for Guangxi cattle. **G**, **H** The expression of circUBE2Q2, MyOD1, MyOG, and MyHC were measured by RT-qPCR of the fetus and at 18 and 24 month-old muscle (**G**), MuSCs, and SVFs (**H**). **I-K** The expression of circUBE2Q2, PPARG, and C/EBPα were measured by rRT-qPCR of the muscle tissue, intramuscular fat, subcutaneous fat (**I** and **J**), and adipogenically differentiated SVFs (**K**). Values are means ± SEMs for three individual animals. ∗ *P* < 0.05, ∗∗∗ *P* < 0.001. Scale bars = 200 μm

In this study, we focused on the role of circRNAs in regulating myogenic differentiation. Since the function of circLMO7 had been confirmed [27], we selected circUBE2Q2 with a higher expression level to explore. CircUBE2Q2 also known as bta_circ_0002760, which was upregulated significantly (by almost 20-fold) in the DM compared with the GM when measured by RT-qPCR (Supplemental file 1, Fig. S9). CircUBE2Q2, located on chromosome 21, is 494 bp in length and is back-spliced by four to nine exons of the UBE2Q2 gene (Fig. 3A). Divergent and convergent primers were designed to characterize CircUBE2Q2 further. After sequencing the PCR products from the RNase R-treated RNA samples, the back-splice junction of CircUBE2Q2 was confirmed (Fig. 3B). These results also confirmed the presence of CircUBE2Q2 in MuSCs. Subsequently, to verify the CircUBE2Q2 expression levels in muscle, RT-qPCR was used to measure its expression in 9 pairs of cattle tissues. The results showed that CircUBE2Q2 was expressed in various tissues, such as muscle, kidneys, and intestines (Fig. 3C). Moreover, fluorescence in situ hybridization results showed that CircUBE2Q2 was mainly located in the cytoplasm (Fig. 3D).

With the increase of age, we found that the area and diameter of muscle fiber per unit of muscle tissue increased and muscle fiber density decreased in Guangxi cattle aged 18 months and 24 months (Fig. 3E, F). RT-qPCR results showed that the expression of muscle growth markers MyoD1, MyoG, and MyHC were decreased in the fetus, 18-month-old, and 24-month-old muscle tissues, and the expression level of CircUBE2Q2 was also decreased (Fig. 3G). It is worth mentioning that compared with MuSCs, CircUBE2Q2 was expressed at a higher level in adipocytes (SVFs) derived from intramuscular fat, while the expression trend of MyoD1, MyoG, and MyHC was opposite to that of CircUBE2Q2 (Fig. 3H). On the other hand, we also detected the muscle tissue, intramuscular fat, and subcutaneous fat of the longissimus dorsi muscle of Guangxi cattle. We found that CircUBE2Q2 and fat development markers PPARG and C/EBPα had the same expression trend, and their expression in intramuscular fat was significantly higher than that in muscle tissue (Fig. 3I). The expression of CircUBE2Q2 in intramuscular fat was significantly higher than that in subcutaneous fat (Fig. 3J). In addition, during the adipogenic differentiation of SVFs, CircUBE2Q2 first increased on the second day (D2). It continued to be expressed until differentiation maturity (D10), similar to the expression of PPARG and C/EBPα (Fig. 3K). These results confirmed that circUBE2Q2 is abundant in muscle, adipose tissue, and cells, suggesting regulating cattle muscle growth and development.

### Effect of CircUBE2Q2 on MuSCs proliferation and apoptosis

**Fig. 4:**
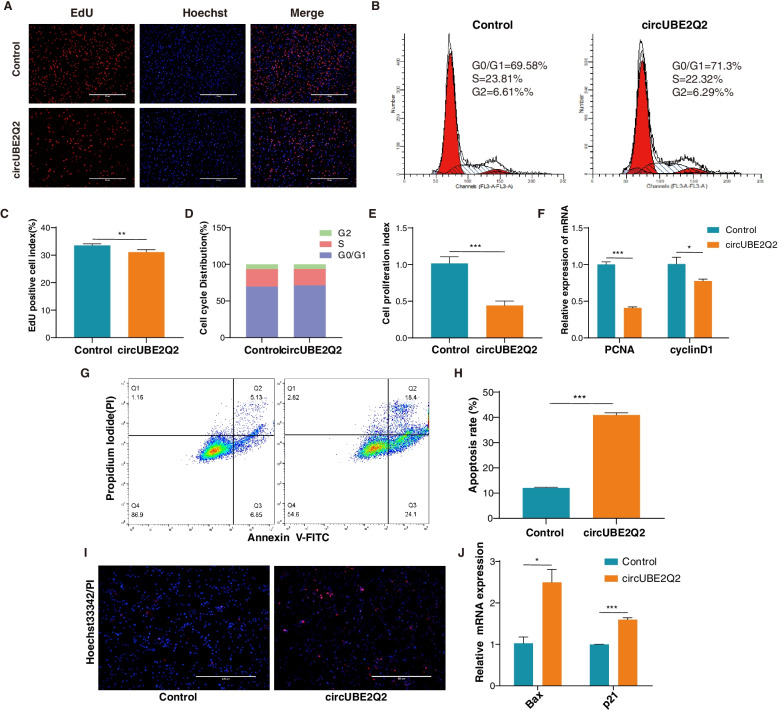
Effect of circUBE2Q2 on proliferation and apoptosis of Guangxi cattle MuSCs. **A** and **C**) Cell proliferation was detected with 5-ethynyl-20-deoxyuridine (EdU)(A), the EdU-positive cell index statistics are shown (**C**). **B** and **D** Cattle MuSCs were transfected with circUBE2Q2, and the cell phase was analyzed (**B**) and counted (**D**) by flow cytometry. **E** Cell proliferation was detected with the cell counting kit-8 (CCK-8) assay. **F** The expression of the proliferation marker genes, PCNA and cyclin D1, were measured by RT-qPCR. **G** and **H** Cattle MuSCs were transfected with circUBE2Q2, and cell apoptosis was determined (**G**) and counted (**H**) by annexin V-FITC/PI binding followed by flow cytometry. **I** Cattle MuSCs were transfected with circUBE2Q2, and cell apoptosis was determined by Hoechst 33342/PI dual staining assays. **J** The mRNA expression of apoptosis marker genes (Bax and p21) by RT-qPCR. Values are means ± SEMs for three individual animals. ∗ *P* < 0.05, ∗*∗ *P* < 0.001. Scale bars = 200/400 μm

To determine the role of CircUBE2Q2 in the proliferation of cattle MuSCs, we transfected pK25-circUBE2Q2 (Supplemental file 1, Fig. S6) into MuSCs to overexpress CircUBE2Q2 significantly (Supplemental file 1, Fig. S5-B). We used EdU, CCK-8, flow cytometry, and RT-qPCR assays to assess its function. First, the results of EdU staining showed that the number of EdU-positive cells had decreased (Fig. 4A, Fig. 4 C). The cell-cycle analysis revealed that overexpression of CircUBE2Q2 did change the proportion of MuSCs in the S-phase and the number of G0/G1 phase cells (Fig. 4B, D). And Cell Counting Kit-8 (CCK-8) assays were found that circUBE2Q2 could inhibit cell viability (*p* < 0.05; Fig. 4E). Furthermore, we also determined the effect of CircUBE2Q2 on the expression of the cell proliferation-related genes such as proliferating cell nuclear antigen (PCNA) and cyclin D1 and found that it was able to decrease the expression of these genes at the mRNA level (*p* < 0.05; Fig. 4F).

Additionally, flow cytometry, Hoechst 33342/PI dual staining apoptosis assays, and RT-qPCR were used to determine the role of CircUBE2Q2 in the apoptosis of cattle MuSCs. Overexpression of CircUBE2Q2 significantly increased the number of apoptotic cells in MuSCs (*p* < 0.05; Fig. 4G, H). In addition, incubation of cattle MuSCs with CircUBE2Q2 increased the number of PI-positive cells after dual staining with Hoechst 33342/PI (Fig. 4I). Finally, we determined the effect of CircUBE2Q2 on cell apoptosis-related genes, Bax and p21, and found that it significantly increased the expression of these genes at the mRNA level (Fig. 4J). Collectively, these results indicated that CircUBE2Q2 promotes MuSCs apoptosis and inhibits MuSCs proliferation.

### Effects of CircUBE2Q2 on differentiation of MuSCs

**Fig. 5:**
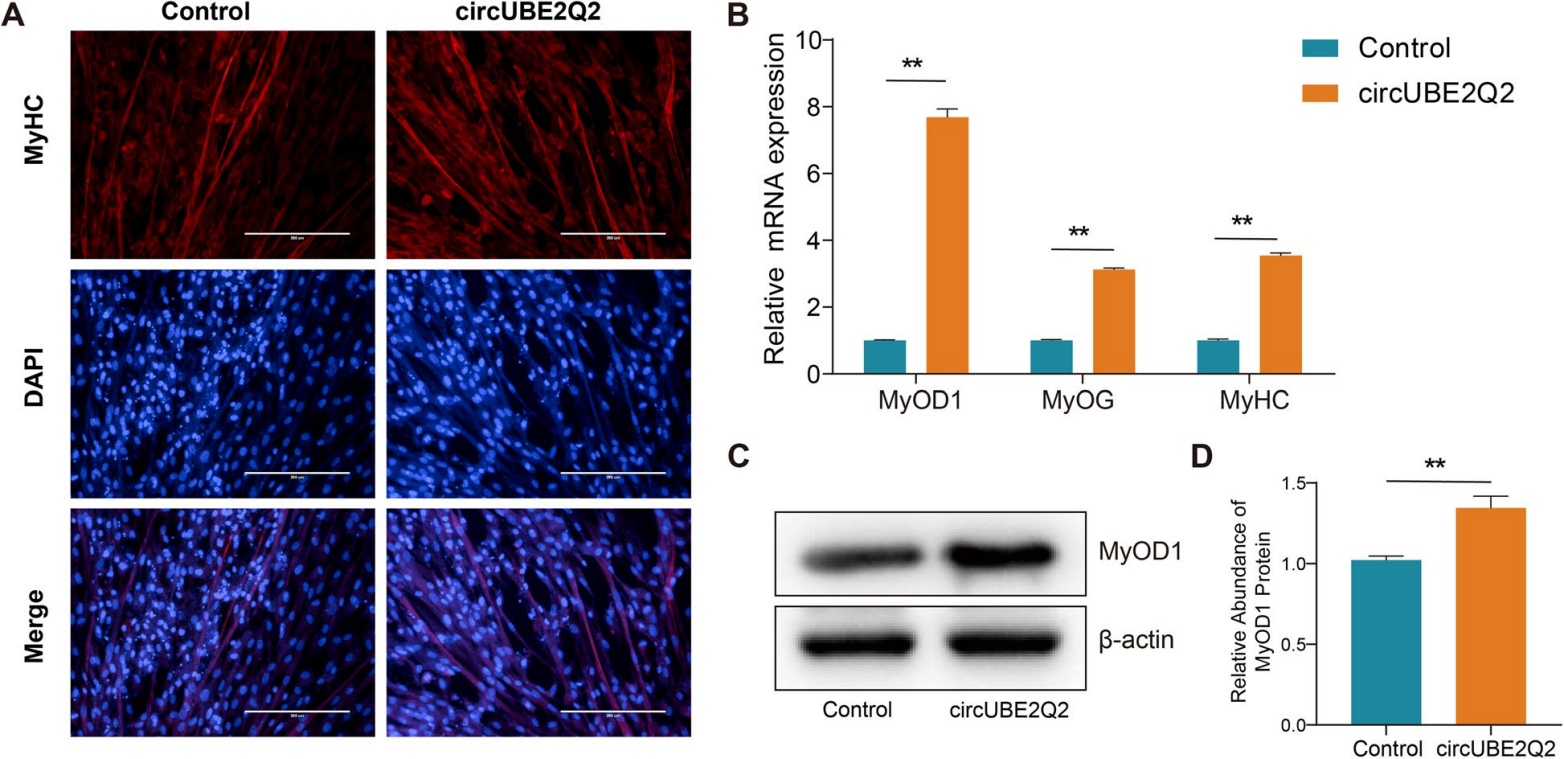
Effects of circUBE2Q2 on differentiation of Guangxi cattle MuSCs. **A** and **B** Cattle MuSCs were transfected with circUBE2Q2, and cell differentiation was detected by immunofluorescence (MyHC) (**A**) and by RT-qPCR (MyOD1, MyOG and MyHC) (**B**). **C** The expression of MyoD1 in primary bovine myoblasts was detected by western blotting. **D** The relative abundance of MyOD1 protein statistics is shown. Values are means ± SEMs for three individual animals. ∗ *P* < 0.05, ∗∗ *P* < 0.01. Scale bars = 100 μm

To investigate the involvement of CircUBE2Q2 in myoblasts differentiation, the expression levels of established myogenic differentiation markers, MyoD1, MyoG, and MyHC, were determined in primary cattle MuSCs treated with CircUBE2Q2 plasmids for 4 days during differentiation. The overexpression of CircUBE2Q2 increased MyHC and myotube formation expression significantly (Fig. 5A). In addition, overexpression of CircUBE2Q2 significantly promoted the expression of myogenic markers MyoD1, MyoG, and MyHC at the mRNA level (Fig. 5B) and the levels of MyoD1 protein (Fig. 5C, D). These findings suggested that CircUBE2Q2 acts to promote Guangxi cattle MuSCs differentiation.

### CircUBE2Q2 serves as a sponge for miR-133a

**Fig. 6:**
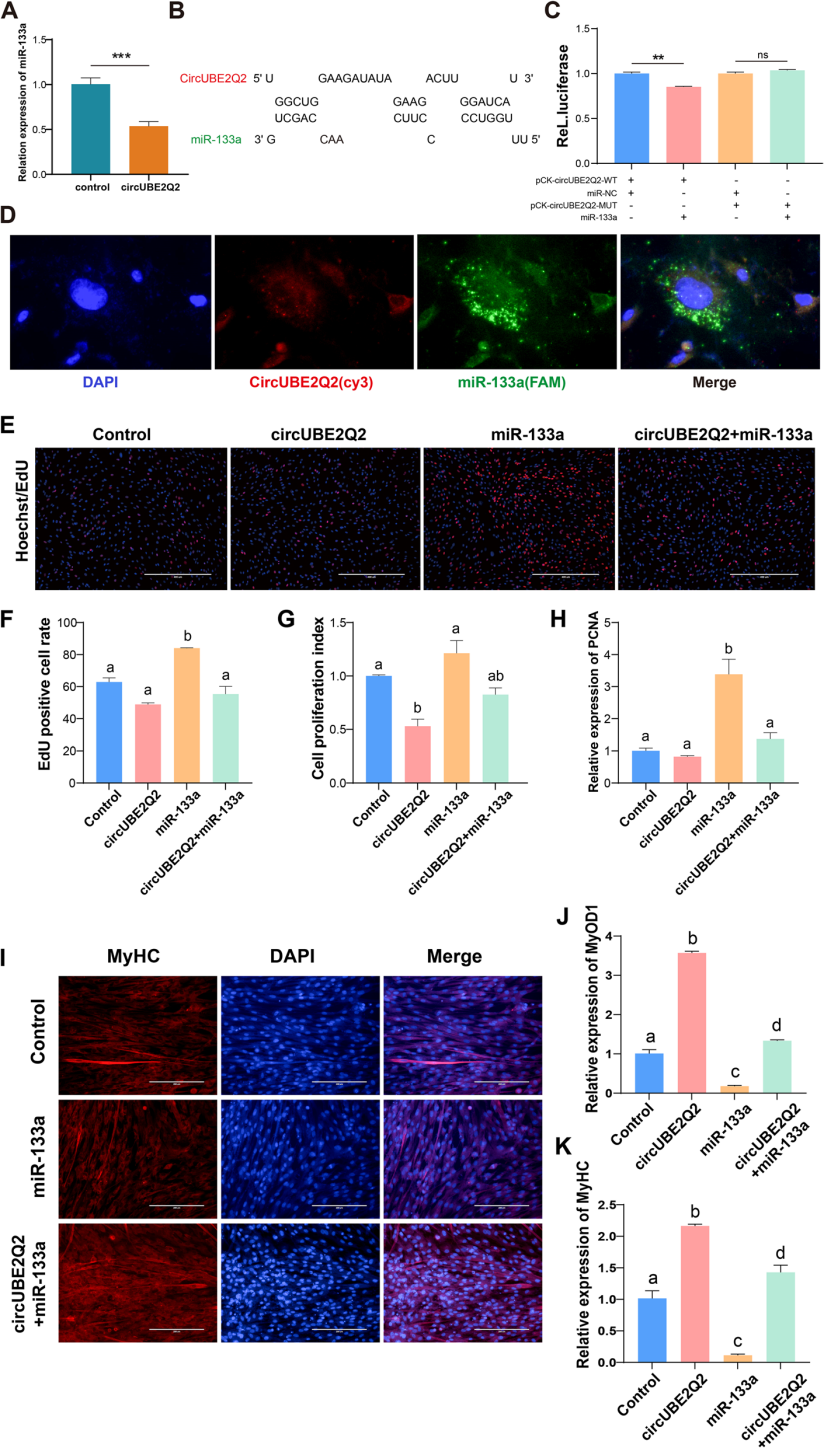
CircUBE2Q2 acts as a sponge for miR-133a. **A** RT-qPCR analysis of miR-133a levels in MuSCs transfected with circUBE2Q2. **B** miR-133a binding sites in the 3′-untranslated region (UTR) of circUBE2Q2 obtained by bioinformatics. **C** Luciferase assay of MuSCs transfected with the psiCHECK2 vector (control) and pCK-circUBE2Q2 reporter with a miR-133a mimic (*n* = 3). **D** Fluorescent in situ hybridization assay shows circUBE2Q2 (red), miR-133a [28], and nuclei (DAPI, blue) in MuSCs. Magnification 73x. **E** MuSCs were transfected with miR-133a and circUBE2Q2, and cell proliferation was measured with 5-ethynyl-20-deoxyuridine (EdU). **F** EdU-positive cell index statistics are shown. **G** Cell proliferation was assessed using the cell counting kit-8 (CCK-8) assay. **H** The expression of the proliferation marker gene, *PCNA*, was measured using RT-qPCR. **I** MuSCs were transfected with the miR-133a and circUBE2Q2, and immunofluorescence detected cell differentiation. **J** and **K** The expression of the myocyte differentiation marker genes MyOD1 (**J**) and MyHC (**K**) were detected by RT-qPCR. Values are means ± SEM. Each letter denotes a significant difference (*P* < 0.05). Scale bars = 100/400 μm

Since CircUBE2Q2 is mainly expressed in the cytoplasm of MuSCs, we evaluated whether CircUBE2Q2 might act as a miRNA sponge to regulate gene expression. We identified 5 candidate miRNAs (miR-133a, miR-133b, miR-1584-5p, miR-2454-3p, and miR-125a) using the predicted miRNA recognition elements overlapping predictions in RNAhybrid to analyze the CircUBE2Q2 sequence (Supplemental file 1, Fig. S7). At present, it has been confirmed that miR-133a is involved in muscle development. First, our results indicated that overexpression of CircUBE2Q2 could significantly reduce miR-133a levels. (Fig. 6A). Moreover, bioinformatics analysis showed that CircUBE2Q2 had miR-133a binding site (Fig. 6B). Moreover, we found that miR-133a was mainly expressed abundantly during the proliferation of MuSCs (Supplemental file 1, Fig. S9). Further, we inserted the entire circUBE2Q2 sequence into the psiCHECK2 luciferase reporter gene (pCK-circUBE2Q2-WT) and constructed its mutant vector (pCK-circUBE2Q2-MUT). The introduction of miR-133a significantly reduced the activity of pCK-circUBE2Q2-WT, however, there was no differential effect on pCK-circUBE2Q2-MUT (Fig. 6C). In addition, FISH detection showed that CircUBE2Q2 and miR-133a co-localized (Fig. 6D). These results supported that circUBE2Q2 acted as a miR-133a sponge. To confirm that CircUBE2Q2 can regulate the level of miR-133a overexpressed MuSCs, we conducted an overexpression study on CircUBE2Q2 in MuSCs. In MuSCs overexpressing miR-133a, cell viability and proliferation rate increased, while overexpression of circUBE2Q2 at the same time, cell viability and proliferation rate decreased (Fig. 6E-G). RT-qPCR confirmed that the administration of miR-133a mimics significantly induced the expression of PCNA (Fig. 6H). On the other hand, in the MuSCs overexpressing miR-133a, the cell differentiation ability was significantly reduced. Myotube fusion and the expression of MYOD1 and MyHC were reduced during myogenic differentiation (Fig. 6I-K).

Together, these results suggested that circUBE2Q2 can serve as a sponge for miR-133a to regulate the proliferation and differentiation of MuSCs.

## Discussion

The growth of muscles in livestock before and early after birth depends on the increase in muscle fibers [29]. When the growth phase ends, muscle fibers no longer increase. However, the volume of the existing fibers continues to increase, affecting meat production capacity and meat quality [30]. Additionally, MuSCs also play an inestimable role in treating muscle diseases, affecting skeletal muscle maintenance, growth, and repair [31, 32].

It is well known that animal MuSCs are responsible for the generation of skeletal muscle during development, accompanied by the dynamic expression of myogenic markers to activate or inhibit their differentiation into a myogenic lineage. This process ultimately maintains tissue homeostasis and repair [33, 34]. This phenomenon suggests that MuSCs are a vital tool for studying the mechanisms that regulate cell fate selection [35, 36].

This study found that MuSCs obtained from Guangxi cattle fetuses successfully underwent a proliferation phase, a growth arrest phase, and a myotube fusion phase. The morphology changed and led to differentiated and fused multi-nucleated mature muscle cells. We used RNA-seq technology to determine the expression of mRNAs, miRNAs, lncRNAs, and circRNAs of Guangxi fetal cattle MuSCs during the proliferation and differentiation phases at the whole transcriptome levels. A total of 19,341 mRNAs were identified and 12,920 non-coding RNAs, including 1011 miRNAs, 8466 lncRNAs, and 3443 circRNAs. In addition, there were 2148 differential mRNAs and 888 non-coding RNAs, including 113 miRNAs, 662 lncRNAs, and 113 circRNAs, during the differentiation of MuSCs from cell proliferation to mature muscle cells. These results enriched the available RNA expression information, which regulates the development of MuSCs in the local Chinese and worldwide beef cattle breeds.

So far, the molecular mechanisms of protein-coding RNAs in cattle muscle still require to be studied [37]. This can provide important reference values for the potential functional mechanism of non-coding RNAs in muscle development. In recent years, the RNA expression profiles of muscle tissues in the embryonic and adult stages of cattle have been established. Many critical regulatory factors such as miRNAs, lncRNAs, and circRNAs have been found [38]. From the perspective of cell proliferation and differentiation, we found that a large number of coding genes were also involved in various biological functional processes of MuSCs development. These differentially expressed coding genes were significantly enriched in 50 signaling pathways, including somatic effects and the cell cycle and TGF-beta and PI3K/Akt signaling pathways. These encompass cell proliferation, invasion, differentiation, autophagy, and other essential cellular processes. The PI3K/Akt/mTOR signaling pathway has emerged as a pivotal regulator of muscle growth and metabolism, and the overexpression of Akt accelerates skeletal muscle hypertrophy [7]. Moreover, MyoD appears to co-operate with the TGFβ/TNFα signaling pathway to regulate skeletal muscle generation [33]. Therefore, these significant signaling pathways will add to our knowledge of myogenesis, and they also provide a solid basis for further analysis of mechanisms controlling muscle mass.

From the results of RT-qPCR, the expression trends of MYF5, IGFN1, MYF6*,* CircCOPB1, and CircXDH were consistent with RNA-seq data, indicating that our data was reliable. In addition, when screening a batch of coding genes such as TP53 as candidate molecules, we further screened candidate molecules in the non-coding RNA database, including the reported miR-1, miR-6 [39], miR-133a [40], and CircLMO7 [27].

As an emerging entry point in RNA research, more and more studies have shown that lncRNAs and circRNAs were thought to become biomarkers for human diseases [41, 42] and animal production [43]. So far, over 170 cellular RNA modifications have been identified in coding and various non-coding RNAs. For example, m6A was considered the most abundant and essential modification in eukaryotic mRNAs, and it regulated mRNA metabolism, including splicing, stability, and translation [44]. M6A modifications also regulated the generation and function of miRNAs, lncRNAs, and circRNAs. However, little is known about m6A modifications to circRNAs [45]. Therefore, in regulating myogenesis, circRNA and miRNA interactions, circRNA translation, circRNA, protein interaction, and m6A modification of circRNAs will focus on our future work.

Combined with the participation of functional genes, myogenic regulatory factors (MRFs), and signaling pathways [46], as well as biological pathways such as cellular mitochondrial respiration [47], the precise regulatory network of circRNAs during MuSCs differentiation was analyzed [48, 49]. Therefore, we conducted a preliminary exploration of the biological functions of CircUBE2Q2. It was found that CircUBE2Q2 and circFGFR4 had similar expression characteristics and functional effects [38]. These had structural stability and were significantly enriched in mature muscle cells. More importantly, CircUBE2Q2 was expressed in multiple tissues, including fetal muscle, kidneys, and liver. The expression of circUBE2Q2 in muscle tissue of cattle decreased with age. It was worth mentioning that circUBE2Q2 was highly expressed in intramuscular fat and upregulated during SVFs adipogenic differentiation. These characteristics suggest that CircUBE2Q2 may regulate muscle development and adipogenesis through homeostatic and metabolic pathways.

Furthermore, CircUBE2Q2 may be an essential gene affecting beef quality and yield traits and is expected to be a candidate gene affecting meat quality traits. Besides, CircUBE2Q2 also serves as a sponge for miR-133a to regulate the proliferation and differentiation of MuSCs. Although the function of CircUBE2Q2 to promote differentiation of cattle MuSCs was identified in this study, the modification pathways and biological processes involved in its ability to regulate muscle cell differentiation need to be further elucidated. Interestingly, previous reports claimed that circUBE2Q2 induced gastric cancer by promoting cell proliferation acceleration [50]. This may be due to factors of different species, cells, and sequences that cause differences in the function of circUBE2Q2. More detailed in vivo and in vitro experiments will be needed to verify the specific roles of CircUBE2Q2 and other candidate circRNAs in muscle development. It may be possible to use the circNfix research model to link these circRNAs with super-enhancers [25, 28, 51, 52] and enrich the internal regulatory network concerning muscle development.

## Conclusion

In conclusion, whole-transcriptome differential expression profiles between MuSCs proliferation and differentiation phases were constructed in this study. DE circUBE2Q2 was characterized and confirmed to be abundant in meat quality-related muscle, adipose tissue, and cells, suggesting its pivotal role in the regulation of beef quality. CircUBE2Q2 can serve as a sponge for miR-133a to regulate the differentiation of MuSCs. The findings in our study will have significant implications to elucidate animal meat production and its quality and establish a potential therapeutic target in the treatment of human muscular diseases.

## Materials and methods

### Sample preparation

At the embryonic stage (90 days), the tissues from Guangxi cattle were collected at a local slaughterhouse in Nanning, Guangxi province. Tissue samples, including muscle, liver, heart, lung, skin, kidney, brain, stomach, and intestine, were collected and immediately frozen in liquid nitrogen. The proliferation of MuSCs was labeled as the GM samples (*n* = 3), and differentiation was then called the DM samples (n = 3). The samples were kept at − 80 °C until RNA was isolated.

### Cell culture

All experiments regarding animals, SVFs were performed in the State Key Laboratory for Conservation and Utilization of Subtropical Agro-bio-resources. They were conducted following its guidelines for the care and use of laboratory animals. Primary cattle MuSCs were isolated and cultured from fetal-derived longissimus muscle as described in Supplemental file 1, using type I collagenase and trypsin combination digestion method. MuSCs were cultured in high-glucose DMEM supplemented with 10–20% fetal bovine serum (FBS; Hyclone, Logan, Utah, USA) and antibiotics (1% penicillin and streptomycin; growth medium (GM)) in an atmosphere of 5% CO_2_ at 37 °C. MuSCs were switched to a differentiation medium (DMEM, 2% horse serum, DM) when cells were almost 90% confluent for up to 4 days to induce MuSCs myogenic differentiation. SVFs were switched to a differentiation medium (10%FBS, 1 μg/ml Dex,0.5 mM IBMX,5 μg/ml insulin), when cells were almost 90% confluent for up to ten days to induce SVFs adipogenic differentiation(Supplemental file 1, Fig. S8).

### Total RNA extraction

The manufacturer’s instructions extracted total RNA from cells and tissues samples with TRizol reagent (Invitrogen, Carlsbad, CA, USA).

### Transcriptome data analysis

Three milligram of RNA per sample was used as the initial starting material for RNA sample preparation. The library was prepared according to the manufacturer’s instructions for using the lncRNA Sample Preparation Kit (Illumina, NEB) and the Small RNA Sample Preparation Kit (Illumina, RS-200-0048) purchased from the Annoroad Gene Technology Corporation (Beijing, China). The circRNA library was based on the lncRNA library, and then Illumina sequencing was performed on the constructed library as described previously [53]. The identification of lncRNAs and circRNAs was identified by following Annoroad’s referenced technical methods. We have provided a detailed description of the methods used in the Supplementary files. All data have been uploaded to the GEO database (accession number: GSE152398).

### Analysis of differentially expressed genes

Differentially expressed (DE) genes, including mRNAs, miRNAs, lncRNAs, and circRNAs, were analyzed using DEseq2. Firstly, the differentially expressed mRNAs and lncRNAs were determined using the corresponding cutoff values (*P* < 0.05, FPKM ≥1, | log2 (fold change) | ≥ 1 for mRNA and *P* < 0.05, FPKM ≥1, | log2 (fold change) | ≥ 1 for lncRNA). Secondly, the differentially expressed miRNAs were determined using the cutoff values of (*P* < 0.05, TPM ≧1, | log2 (fold change) |≧1). Finally, the expression levels of the circRNAs were normalized by spliced reads per billion mapped (SRPBM). *P* < 0.05, SRPBM≥1 and |log2 (fold change)| ≥ 1 was set as the threshold for determination of the significant differential expression levels of the circRNAs.

### Gene ontology (GO) and KEGG analysis

GO (http://www.geneontology.org) and KEGG pathway (http://www.kegg.jp) were analyzed as described previously [54–56].

### Quantitative real-time RT-PCR

According to the manufacturer’s instructions, total RNA was extracted using TRizol reagent (Invitrogen, Carlsbad, CA, USA). Reverse transcription was performed using the HiScript R II One-Step RT-PCR kit (Vazyme, Nanjing, China). RT-qPCR was carried out using the ChamQ SYBR qPCR Master Mix (Vazyme, Nanjing, China) with β-actin as the internal control and the 2^-ΔΔCt^ method. All primer sequences used are shown in Supplemental file 2 (Table S2a-S2 b).

### RNase R treatment and validation of circRNAs

Ribosome-depleted RNAs were incubated for 15 min at 37 °C with 5 units RNase R per μg RNA (Epicentre Technologies, Madison, WI, USA). RT-PCR and sequencing assays were performed as described previously [57], and circUBE2Q2 was validated. All primers sequences used are shown in Supplemental file 2 (Table S2c).

### Vector construction

A molecular biology company completed the construction and sequencing identification of circRNA overexpression vectors (Geneseed, Guangzhou, China) (Supplemental file 1, Fig. S5-A). The whole length of circUBE2Q2 was cloned into pK25ssAAV-ciR (Geneseed, Guangzhou, China) to obtain an expression plasmid (pK25-circUBE2Q2). All primer sequences used are shown in Supplemental file 2 (Table S2d).

### Cells treatment

MuSCs were grown to 70% confluence and then trypsinized and plated at 5 × 10^5^ cells/well into 6-well plates and 1 × 10^4^ cells/well in 96-well plates (Thermo Fisher Scientific, Waltham, MA, USA) and incubated as described previously [17]. They were then transfected with pK25-circUBE2Q2 (overexpression group) and pK25ssAAV-ciR (control group) using X-treme GENE HP DNA Transfection Reagent (Roche, Basel, Switzerland). After incubation, the MuSCs were used for the different assays outlined below. The culture medium was changed to high-glucose DM to induce differentiation of myoblasts.

### 5-Ethynyl-20-deoxyuridine (EdU) assay

According to the manufacturer’s instructions, a cell-Light EdU DNA cell proliferation kit was used to assess cell proliferation, and each treatment group had three independent replicates.

### Hoechst 33342 and propidium iodide (PI) dual staining assays

Hoechst 33342 and PI double staining (Solarbio, Beijing, China) measured cell apoptosis. According to the kit’s requirements, cells were incubated with Hoechst 33342 for 20 min at room temperature. Then the cells were treated with PI for 10 min at room temperature. The fluorescence signal was assessed using a fluorescence microscope (Nikon).

### Flow cytometry

The cell cycle of different treatment groups was measured using the cell cycle testing kit (Multisciences, Hangzhou, China). After incubation, the cell suspension obtained was used for flow cytometry (FACS Canto II, BD BioSciences, USA), and each treatment group had three independent replicates. In a flow cytometer, the cell apoptosis of MuSCs was also measured by flow cytometry using FITC-labeled Annexin V and propidium iodide (PI).

### Immunofluorescence and microscopy

MuSCs were washed three times with PBS buffer (pH 7.4) and permeabilized for 15 min in PBS containing 0.5% Triton X-100 before fixation in PBS containing 4% paraformaldehyde for 20–30 min. Immunostaining was carried out as follows: cells were incubated overnight at 4 °C with the primary anti-PAX7 (1:50, Proteintech) anti-MyoD1 (1:200; Abcam) and anti-MyHC (1:100; Abcam) antibodies, diluted in 5% bovine serum albumin. After that, cells were washed with PBS and incubated at room temperature for 3 h with the corresponding secondary antibodies, goat anti-rabbit IgG (H + L; 1:1000; Invitrogen) or goat anti-mouse IgG (H + L;1:1000; Invitrogen) diluted in PBS. DNA was visualized using 5 mg/mL DAPI staining. Finally, the prepared cells were washed four times with PBS and observed under a fluorescence microscope (Nikon).

### Western blotting

Cells were collected from different treatment groups, pelleted by centrifugation, and then lysed in RIPA buffer. Total protein was prepared, and protein concentrations were determined using the Bradford method. Proteins were then separated by SDS-polyacrylamide gel electrophoresis (SDS-PAGE) and subsequently transferred to nitrocellulose membranes and blocked with 5% skimmed milk powder solution for 1.5–2 h at room temperature. The membranes were then incubated overnight with the primary antibodies. Anti-MyOD1 and anti-β-actin were purchased from Abcam (Cambridge, MA, USA). After that, the membranes were washed with PBS-tween and incubated for a further 1.5 h with horseradish peroxidase-conjugated secondary antibodies (Abcam, Cambridge, MA, USA). After treatment, protein bands were detected with Super Signal West Femto reagent purchased from Thermo (Thermo Scientific, Waltham, MA, USA).

### Dual-luciferase reporter system assay

Luciferase activity was validated using HEK293T cells. First, wild-type circUBE2Q2 luciferase reporter plasmids and miRNA mimics were transfected into 293 T cells, and their target relationships were analyzed. Secondly, the Dual-Luciferase Reporter (DLR) Assay System Kit (Promega, Madison, WI, USA) was then used to measure the luciferase activity according to the manufacturer’s instructions.

### Fluorescence in situ hybridization

Fluorescence in situ hybridization (FISH) was performed using in situ hybridization reagents (Servicebio, Wuhan, China). First, MuSCs were cultured on glass slides and incubated with FISH fixative for 30–40 min at room temperature. Second, they were incubated with PBS containing 0.2% Triton X-100 at room temperature for 5 min to penetrate the cell membrane and then incubated with the pre-hybridization solution at 37 °C for 1 h. Then, the hybridization solution of fixed cells and fluorescently labeled circUBE2Q2 and miR-133a probe were placed in a black wet box and incubated overnight at 4 °C. Next, the cells were washed with PBS solution five times, and 40,6-diamino-2-phenylindole (DAPI, 1:1000) solution was added and incubated for 5 min at room temperature in the dark. The whole experiment requires the slides to be placed in a humid box to keep the cells moist. Finally, a fluorescence microscope (Nikon, Tokyo, Japan) was used for cell observation, and fluorescence photos were taken.

### Statistical analysis

The quantitative results are presented as mean ± SEM based on at least three independent experiments. Significant variations by treatments in comparison to the untreated samples were determined by one-way ANOVA performed with GraphPad Prism version 8.0 (GraphPad Software, La Jolla, CA, USA). Differences were considered significant when *P*-values ≤0.05 were obtained.

## Supplementary Information

**Additional file 1: Figure S1.** Isolation, culture and differentiation of Guangxi cattle muscle stem cells (A) Cattle fetus around 3 months old. (B) Primary muscle stem cells cultured in vitro for 48 hours. (C) GM sample (proliferation) of MuSCs. (D) DM sample (differentiation) of MuSCs. (scale bars = 100/200 μm). **Figure S2.** The workflow of RNA-seq. **Figure S3.** Cluster analysis of differentially expressed RNA in MuSCs.(A–D) Volcano plots (below) displaying the differentially expressed transcripts and the hierarchical cluster analysis is shown above of each panel which displays the differential expression of RNAs in three DM samples and three GM samples of MuSCs. (A) mRNAs, (B) miRNAs, (C) lncRNAs and (D) circRNAs. The blue and yellow dots represent downregulated and upregulated RNAs in DM of MuSCs respectively, when compared with GM. The grey dots indicate no significant difference. **Figure S4.** Characteristics of circular RNA in MuSCs of Guangxi Cattle. (A) Distribution of genomic regions from where the detected circRNAs were derived. (B) Chromosomal distribution of circRNAs. (C) Distribution of the number of circRNAs per gene. (D) Distribution of sample expression for circRNAs. (E) Length distribution of cricRNAs. (F) SRPBM distribution of circRNAs. **Figure S5-B.** Cell transfection of overexpression vector pK25-circUBE2Q2 and visualization of the efficiency of circUBE2Q2. **Figure S6.** Vector construction of plasmids. **Figure S7.** Identification results of candidate miRNAs. **Figure S8.** The culture and adipogenic differentiation of SVFs. **Figure S9.** Validation of RNAs identified from RNA-seq in cattle MuSCs. **Table S1.** The information of differential RNAs. **Table S2. a** primers for RT-PCR. **b** primers for RT-qPCR. **c** primers for PCR. d primers for vector construction.

## Data Availability

All raw transcriptome data supporting the conclusions of this article is available in GEO database (accession number: GSE152398).

## Abbreviations

MuSCs: muscle stem cells
MRFs: Myogenic regulatory factors
RNase R: Ribonuclease R
DE: Differentially expressed
FPKM: Fragments Per Kilobase of exon model per Million mapped fragments
TPM: Transcripts Per Million
SRPBM: Spliced reads per billion mapped
GM: Growth medium
DM: Differentiation medium
