# Supplementary material for: CircUBE2Q2 promotes differentiation of cattle muscle stem cells and is a potential regulatory molecule of skeletal muscle development
Source: BMC Genomics. 2022 Apr 6;23:267. doi: 10.1186/s12864-022-08518-4 (PMC8985345; doi:10.1186/s12864-022-08518-4)
Supplement: Supplementary file 1 — Additional file 1: Figure S1. Isolation, culture and differentiation of Guangxi cattle muscle stem cells (A) Cattle fetus around 3 months old. (B) Primary muscle stem cells cultured in vitro for 48 hours. (C) GM sample (proliferation) of MuSCs. (D) DM sample (differentiation) of MuSCs. (scale bars = 100/200 μm). Figure S2. The workflow of RNA-seq. Figure S3. Cluster analysis of differentially expressed RNA in MuSCs.(A–D) Volcano plots (below) displaying the differentially expressed transcripts and the hierarchical cluster analysis is shown above of each panel which displays the differential expression of RNAs in three DM samples and three GM samples of MuSCs. (A) mRNAs, (B) miRNAs, (C) lncRNAs and (D) circRNAs. The blue and yellow dots represent downregulated and upregulated RNAs in DM of MuSCs respectively, when compared with GM. The grey dots indicate no significant difference. Figure S4. Characteristics of circular RNA in MuSCs of Guangxi Cattle. (A) Distribution of genomic regions from where the detected circRNAs were derived. (B) Chromosomal distribution of circRNAs. (C) Distribution of the number of circRNAs per gene. (D) Distribution of sample expression for circRNAs. (E) Length distribution of cricRNAs. (F) SRPBM distribution of circRNAs. Figure S5-B. Cell transfection of overexpression vector pK25-circUBE2Q2 and visualization of the efficiency of circUBE2Q2. Figure S6. Vector construction of plasmids. Figure S7. Identification results of candidate miRNAs. Figure S8. The culture and adipogenic differentiation of SVFs. Figure S9. Validation of RNAs identified from RNA-seq in cattle MuSCs. Table S1. The information of differential RNAs. Table S2. a primers for RT-PCR. b primers for RT-qPCR. c primers for PCR. d primers for vector construction. [file 12864_2022_8518_MOESM1_ESM.zip › Revison of Supplemental file 1.docx]

1. Isolation of muscle stem cells

We used 0.2% Roche type I collagenase digestion method to isolate muscle stem cells derived from the dorsal longest muscle of fetuses around 3 months old to establish cell lines: (1) Collect muscle tissue: treated the muscle tissue separately with sterilized ultrapure water, 75% alcohol and PBS; (2) Shred tissue: cut the tissue into 1mm^3^ fragments; (3) Digestion tissue: first digest the tissue with 0.2% Roche type I collagenase for 1h, then digest with 0.25% pancreatin for 25 min, and stop the digestion; (4) Harvest the cells: filter the cells with a 70 um sieve 1-2 times to obtain the cells; (5) Cell culture: cells are cultured in 37°C, 5% CO2 environment; (6) Purified cells: after 2 hours of culture, transfer the cell liquid to a new Petri dish to continue culturing, and repeat this step 2 times to continue culturing.


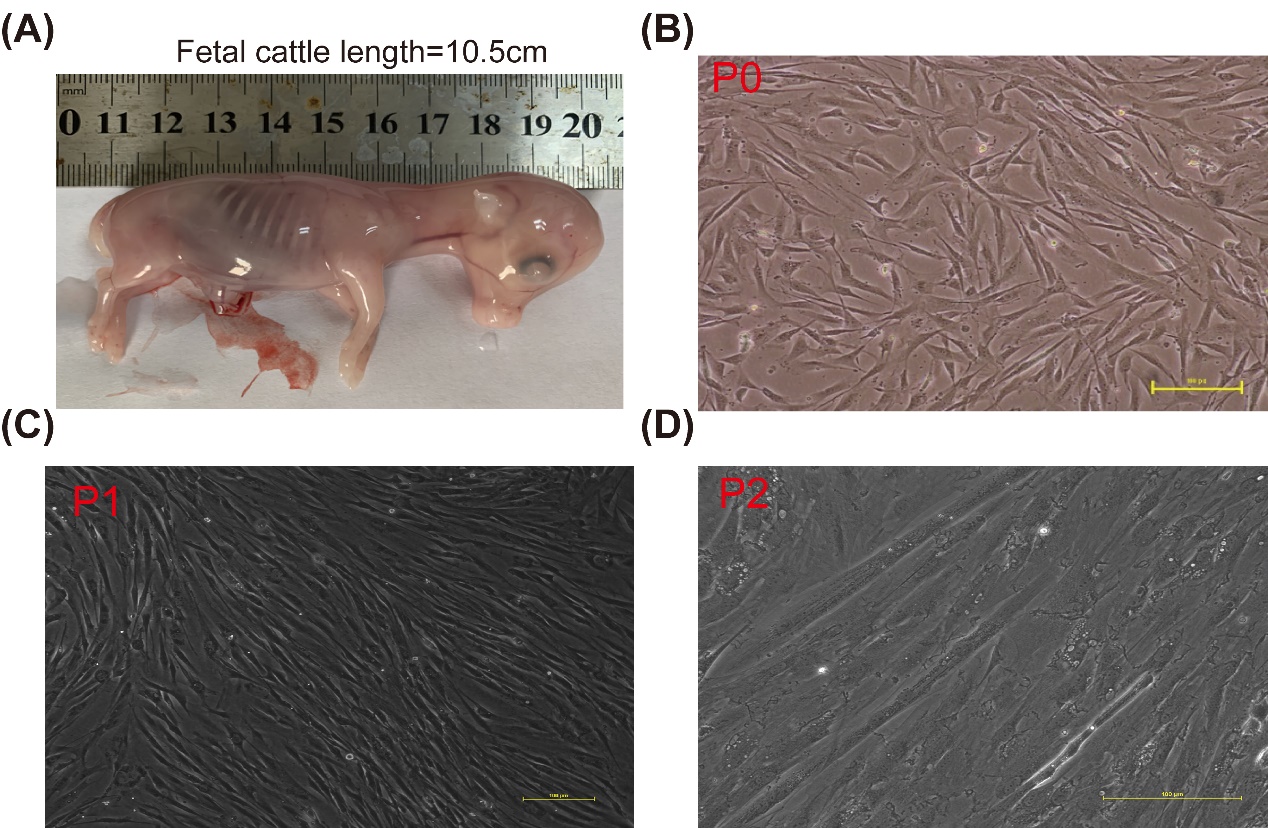


Figure S1. Isolation, culture and differentiation of Guangxi cattle muscle stem cells (A) Cattle fetus around 3 months old. (B) Primary muscle stem cells cultured in vitro for 48 hours. (C) GM sample (proliferation) of MuSCs. (D) DM sample (differentiation) of MuSCs. (scale bars = 100/200 μm).

1. RNA-seq analysis of MuSCs samples


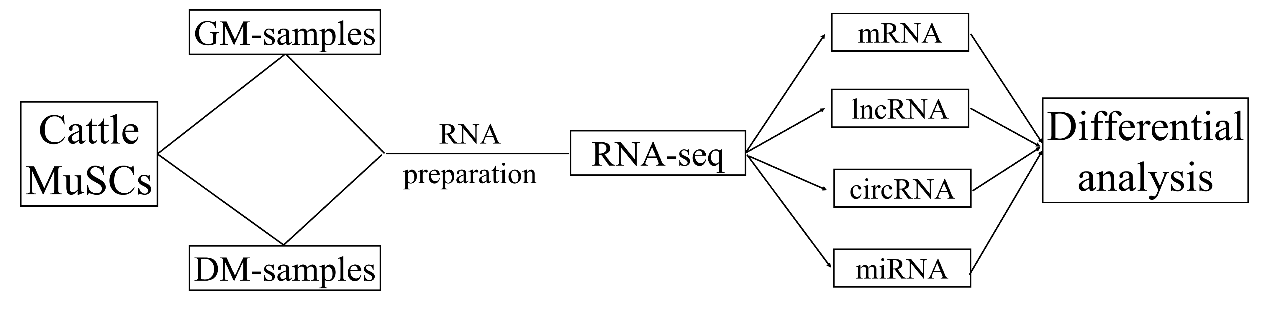


Figure S2. The workflow of RNA-seq.

3. Characteristics of differentially expressed RNA


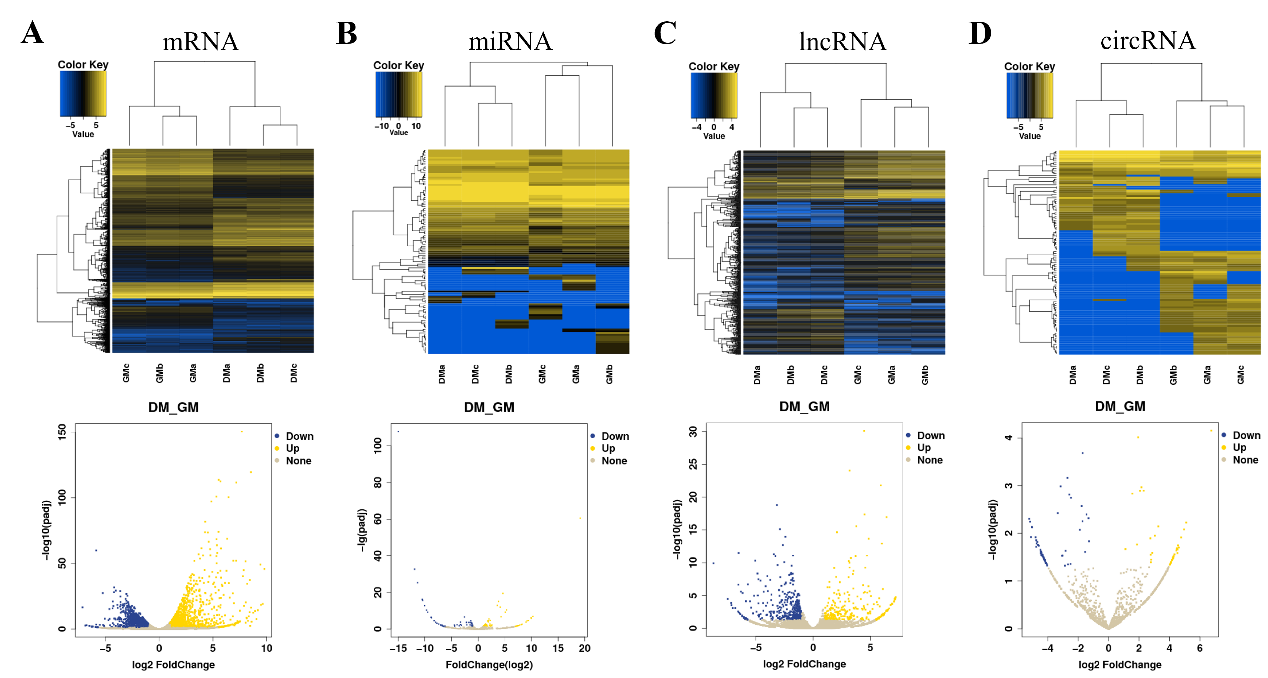


Figure S3. Cluster analysis of differentially expressed RNA in MuSCs.(A–D) Volcano plots (below) displaying the differentially expressed transcripts and the hierarchical cluster analysis is shown above of each panel which displays the differential expression of RNAs in three DM samples and three GM samples of MuSCs. (A) mRNAs, (B) miRNAs, (C) lncRNAs and (D) circRNAs. The blue and yellow dots represent downregulated and upregulated RNAs in DM of MuSCs respectively, when compared with GM. The grey dots indicate no significant difference.

4. Characteristics of circular RNA


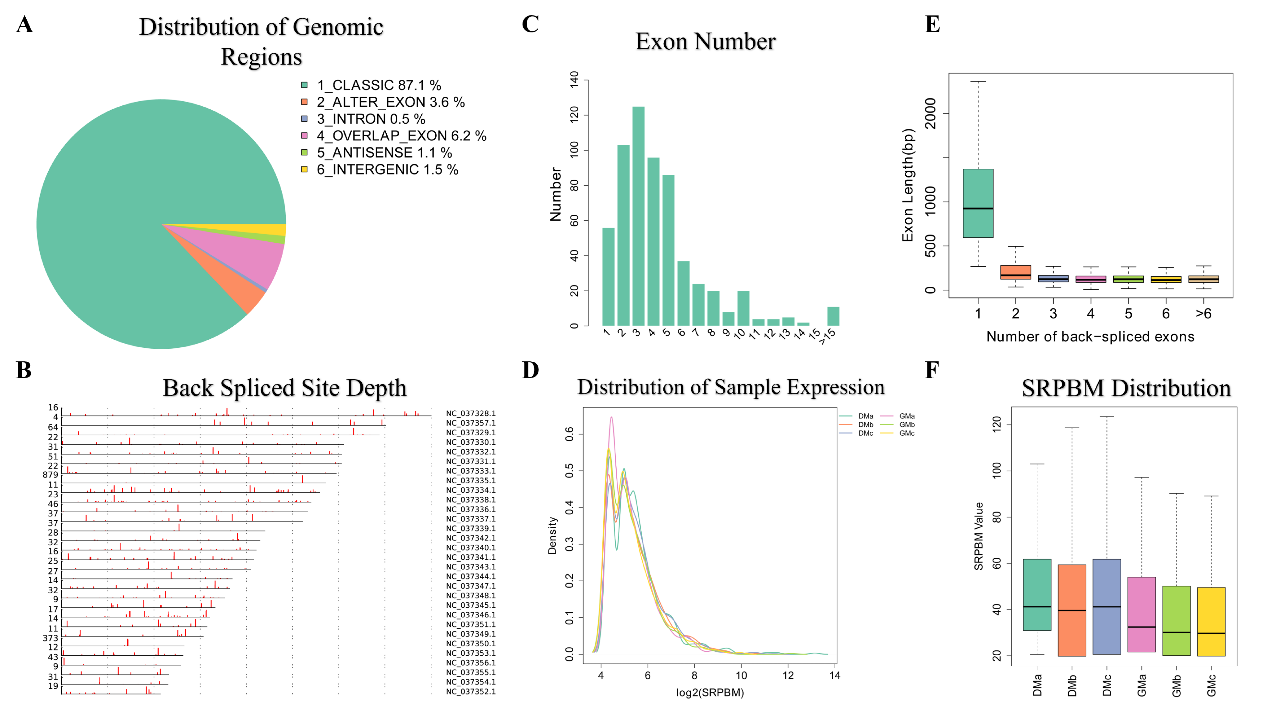


Figure S4. Characteristics of circular RNA in MuSCs of Guangxi Cattle. (A) Distribution of genomic regions from where the detected circRNAs were derived. (B) Chromosomal distribution of circRNAs. (C) Distribution of the number of circRNAs per gene. (D) Distribution of sample expression for circRNAs. (E) Length distribution of cricRNAs. (F) SRPBM distribution of circRNAs.

5. Vector construction

The whole length of circUBE2Q2 was cloned into pK25ssAAV-ciR (5765bp) to obtain their expression plasmids (pK25-circUBE2Q2,6259bp).


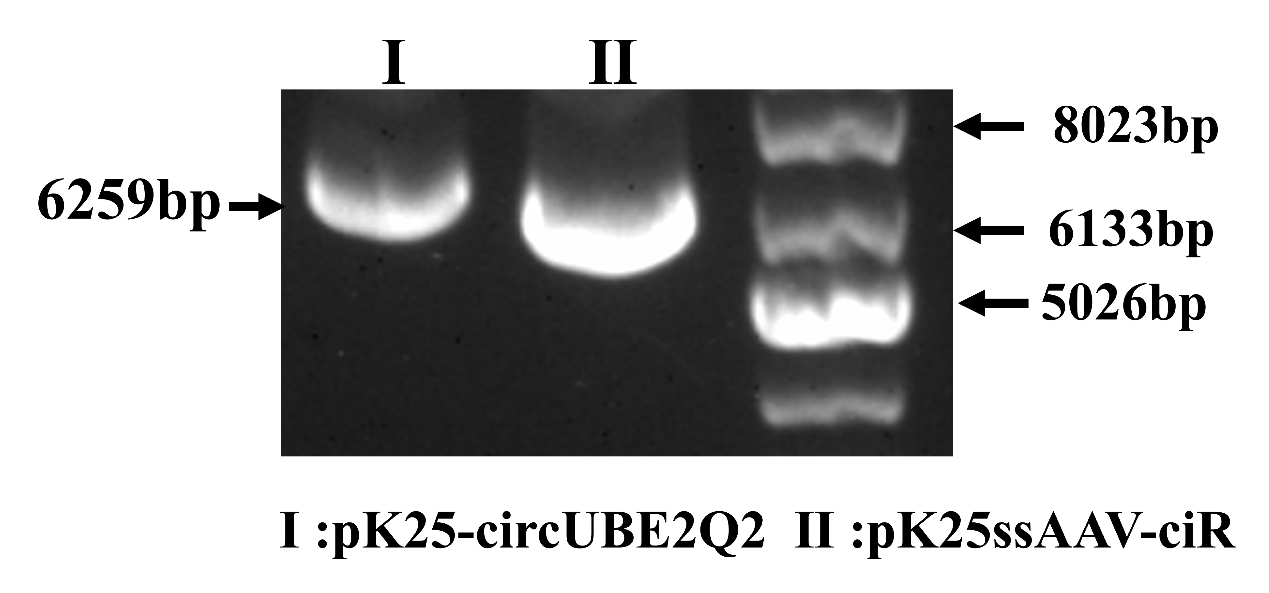


Figure S5-A. Vector construction of circUBE2Q2


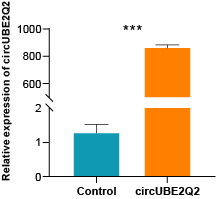


Figure S5-B. Cell transfection of overexpression vector pK25-circUBE2Q2 and visualization of the efficiency of circUBE2Q2.

6. Cells transfection

MuSCs were transfected with pK25-circUBE2Q2 (overexpression group) and pK25ssAAV-ciR (control group) plasmids. After 24 hours, the reporter gene green fluorescence protein (GFP) was expressed strongly in the transfected cells.


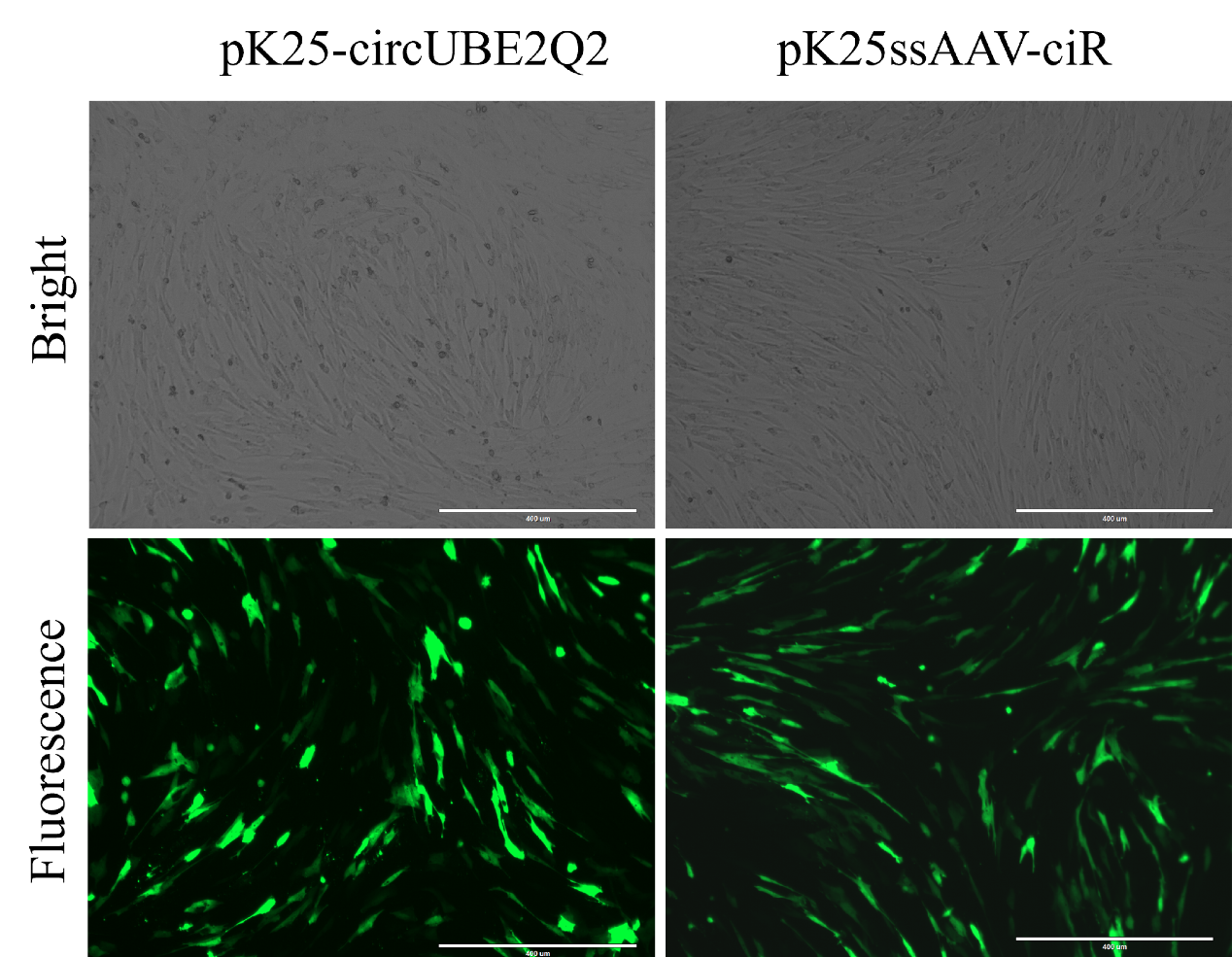


Figure S6. Vector construction of plasmids

7. Identification results of candidate miRNAs


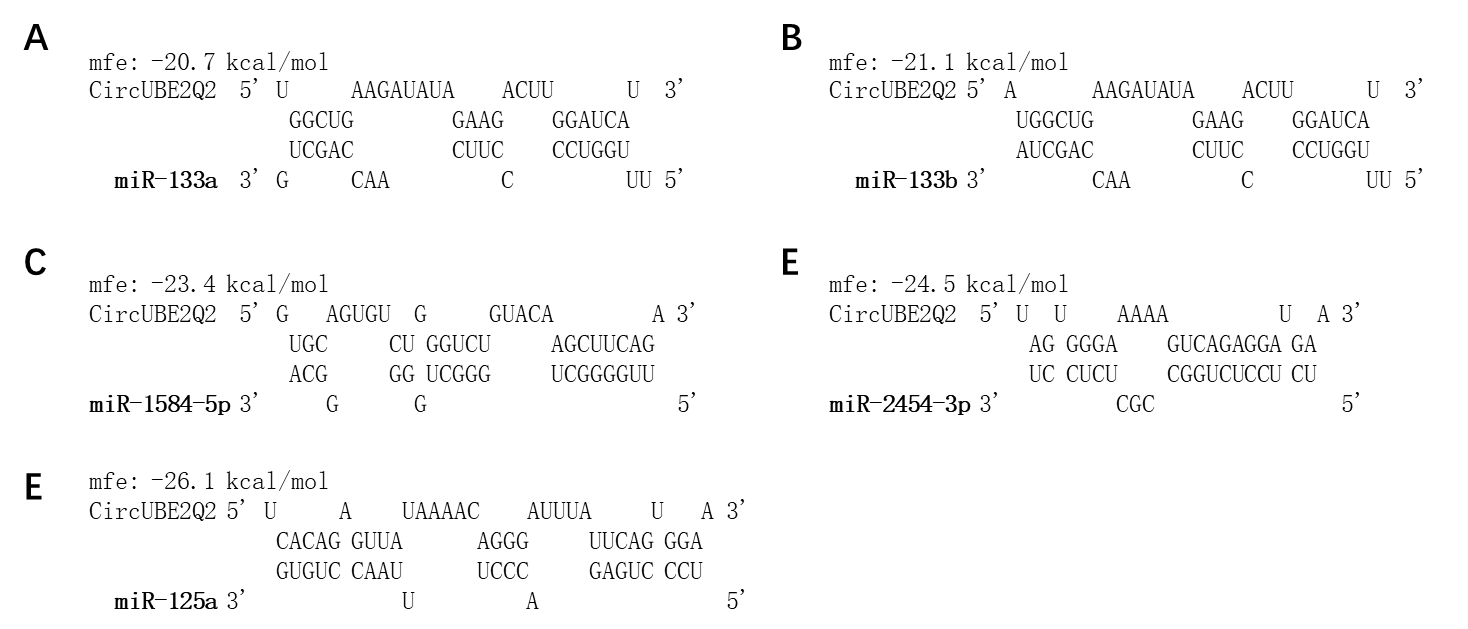


Figure S7. Identification results of candidate miRNAs.

8. The culture and adipogenic differentiation of stromal vascular fraction cells (SVFs).


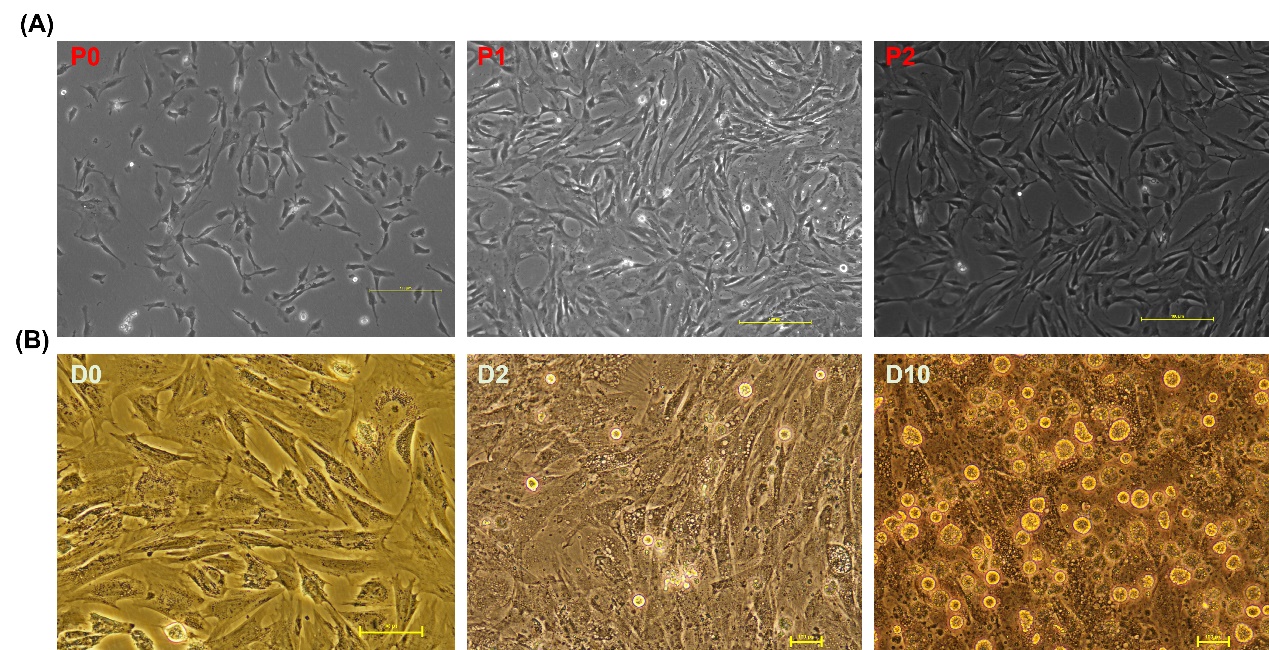


Figure S8. The culture and adipogenic differentiation of SVFs.

9. Validation of RNAs identified from RNA-seq in cattle MuSCs by real-time quantitative PCR.

The validation results of mRNAs, miRNAs, lncRNAs and circRNAs. * P < 0.05, ∗∗ P < 0.01 and ∗∗∗ P < 0.001.


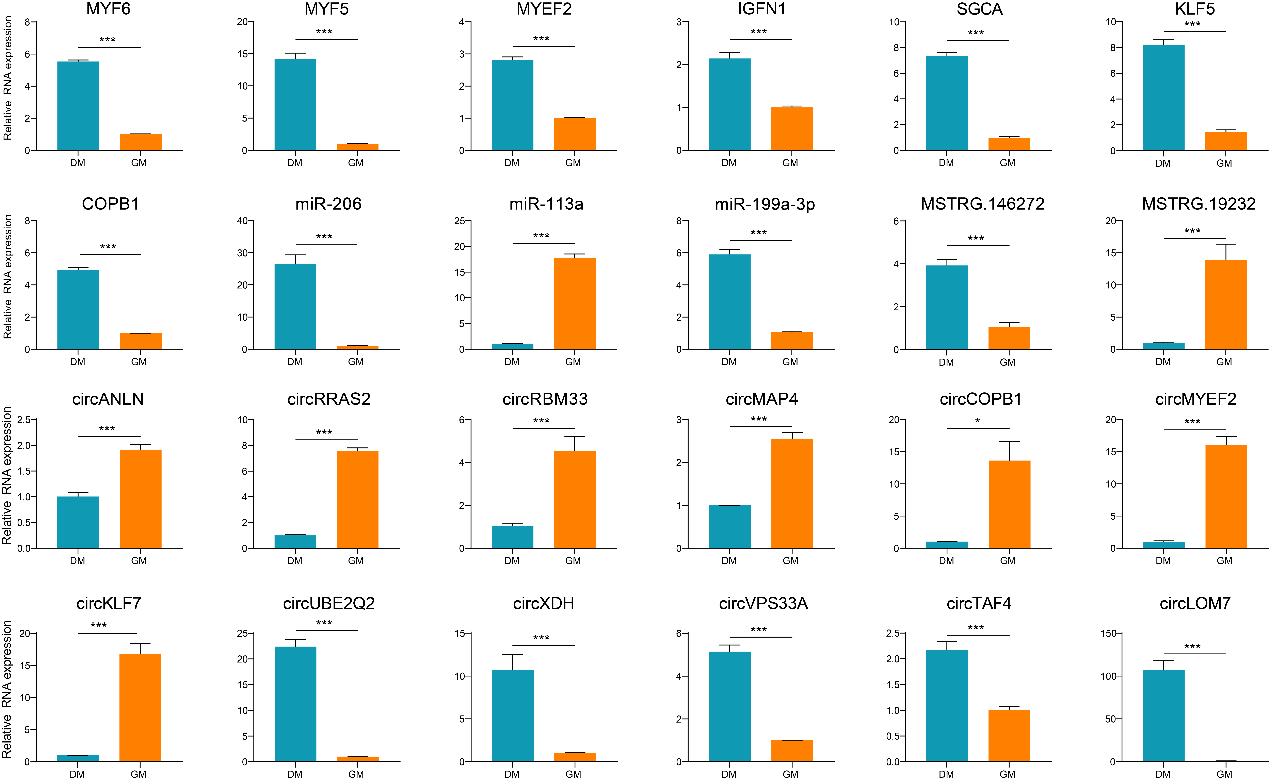


Figure S9. Validation of RNAs identified from RNA-seq in cattle MuSCs
