# Supplementary figures and images for: CircUBE2Q2 promotes differentiation of cattle muscle stem cells and is a potential regulatory molecule of skeletal muscle development
Source: BMC Genomics. 2022 Apr 6;23:267. doi: 10.1186/s12864-022-08518-4 (PMC8985345; doi:10.1186/s12864-022-08518-4)

circUBE2Q2





vector construction


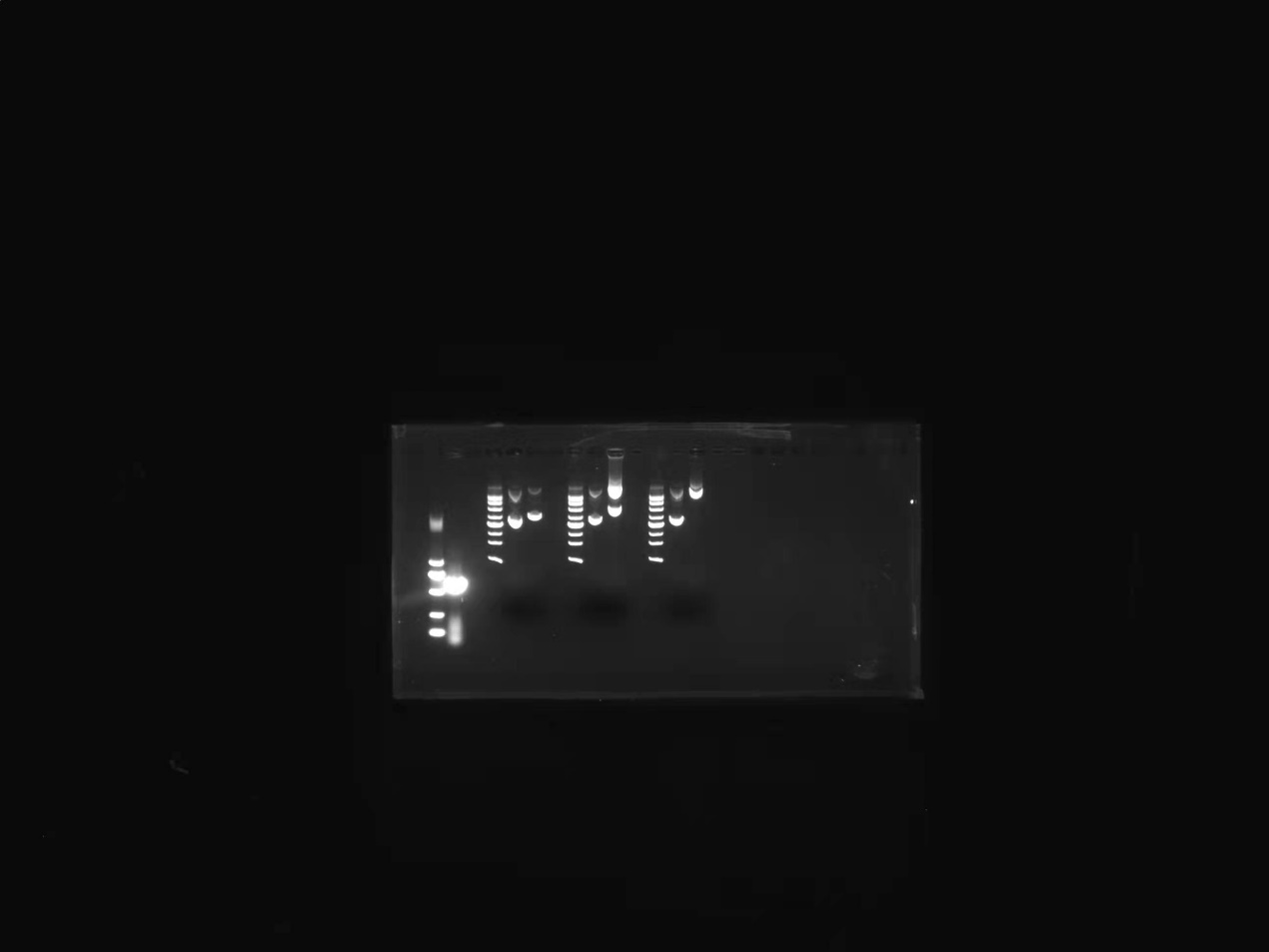


WB

MyOD1


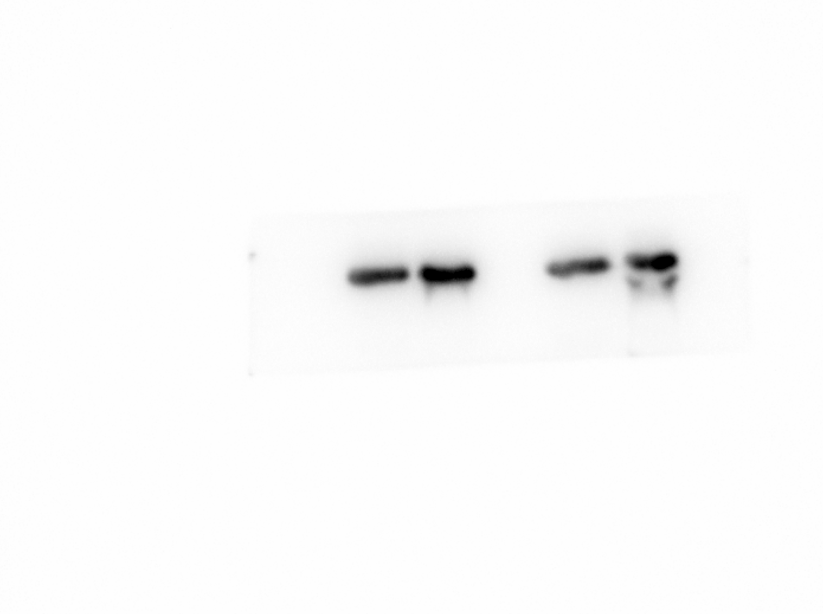


β-actin


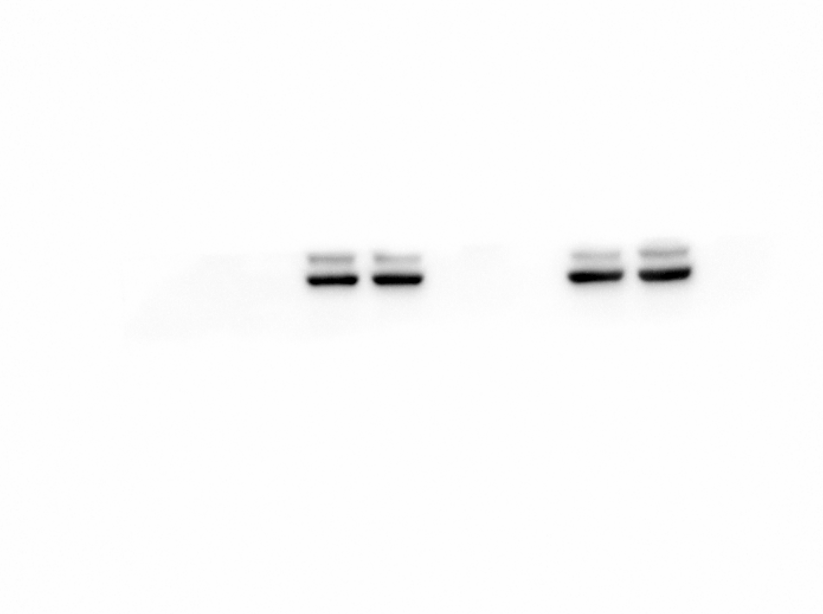

Supplement: Supplementary file 1 — Additional file 1: Figure S1. Isolation, culture and differentiation of Guangxi cattle muscle stem cells (A) Cattle fetus around 3 months old. (B) Primary muscle stem cells cultured in vitro for 48 hours. (C) GM sample (proliferation) of MuSCs. (D) DM sample (differentiation) of MuSCs. (scale bars = 100/200 μm). Figure S2. The workflow of RNA-seq. Figure S3. Cluster analysis of differentially expressed RNA in MuSCs.(A–D) Volcano plots (below) displaying the differentially expressed transcripts and the hierarchical cluster analysis is shown above of each panel which displays the differential expression of RNAs in three DM samples and three GM samples of MuSCs. (A) mRNAs, (B) miRNAs, (C) lncRNAs and (D) circRNAs. The blue and yellow dots represent downregulated and upregulated RNAs in DM of MuSCs respectively, when compared with GM. The grey dots indicate no significant difference. Figure S4. Characteristics of circular RNA in MuSCs of Guangxi Cattle. (A) Distribution of genomic regions from where the detected circRNAs were derived. (B) Chromosomal distribution of circRNAs. (C) Distribution of the number of circRNAs per gene. (D) Distribution of sample expression for circRNAs. (E) Length distribution of cricRNAs. (F) SRPBM distribution of circRNAs. Figure S5-B. Cell transfection of overexpression vector pK25-circUBE2Q2 and visualization of the efficiency of circUBE2Q2. Figure S6. Vector construction of plasmids. Figure S7. Identification results of candidate miRNAs. Figure S8. The culture and adipogenic differentiation of SVFs. Figure S9. Validation of RNAs identified from RNA-seq in cattle MuSCs. Table S1. The information of differential RNAs. Table S2. a primers for RT-PCR. b primers for RT-qPCR. c primers for PCR. d primers for vector construction. [file 12864_2022_8518_MOESM1_ESM.zip › Full scan of Gel and Western blots original data.docx]
